# The evolutionary journey to a new normal for learning and capacity building of healthcare workers to prepare and respond to health emergencies across Africa

**DOI:** 10.3389/fpubh.2024.1455444

**Published:** 2025-01-21

**Authors:** Boukaré Bonkoungou, Fausta Mosha, Amarachi Abianuru, Joseph Okeibunor, Heini Utunen, Giselle Balaciano, Ana C. Barbosa de Lima, Lauren Burke, Shannon McKenna, Sukriti Nag, Abdou Salam Gueye, Bruce Struminger

**Affiliations:** ^1^World Health Organization, Brazzaville, Republic of Congo; ^2^World Health Organization, Geneva, Switzerland; ^3^Department of Internal Medicine, ECHO Institute, University of New Mexico Health Sciences Center, Albuquerque, NM, United States; ^4^ECHO India Trust, New Delhi, India

**Keywords:** health emergencies, capacity building, learning interventions, health information, just-in-time learning, knowledge dissemination

## Abstract

**Background:**

Faced with the COVID-19 mobility restrictions, the WHO AFRO EPR program pioneered a collaborative initiative with Project ECHO to virtually educate health workers across Africa at scale. This initiative has evolved into a transformative learning journey. This paper aims to present the lessons learned.

**Results:**

From April 2020 to February 2024, 287 virtual learning sessions were conducted with over 125,816 attendances from 173 countries and regions. This marked a significant increase compared to pre-pandemic face-to-face training, which targeted fewer than 2,000 participants annually. Survey responses (*n* = 43,221) indicated high relevance and applicability, with 97% of respondents planning to use the information in their work and 89% finding the sessions very or extremely relevant. Self-reported knowledge levels increased from 28 to 74% post-session.

**Discussion:**

Integrating digital learning into WHO AFRO's education and training toolkit has facilitated immediate emergency responses and enhanced long-term resilience, adaptability, and equity among healthcare workers, especially in under-resourced regions. This initiative has reached more health professionals than pre-pandemic in-person training, facilitating more equitable access to essential knowledge and best practices.

**Conclusion:**

The WHO AFRO and Project ECHO partnership navigated a variety of challenges, while establishing a paradigm shift in learning strategies. Emphasizing a digital learning first approach, while retaining in-person elements, this collaborative initiative offers insights for future healthcare education, highlighting adaptability, cost-efficiency, equity, and new technologies in addressing global health challenges. However, to sustain this momentum and further expand access to critical knowledge, stakeholders must commit to continued investment in digital learning infrastructure, training, and technology.

## 1 Introduction

The onset of the COVID-19 pandemic led to unprecedented disruptions in global health systems, prompting an urgent reevaluation of the methods used to train healthcare workers (1). Traditional in person training methods were not possible to implement due to travel restrictions and there was a need for rapid, widespread dissemination of the latest health guidance and practices. In response to these challenges, the World Health Organization Regional Office for Africa (WHO AFRO) in partnership with Project ECHO hosted by the University of New Mexico (UNM) and the ECHO India Trust, embarked on an innovative initiative to train health workers across Africa through synchronous virtual learning approaches. This venture, initially a temporary solution to the pandemic's constraints, has since matured into a transformative journey of healthcare worker continuing professional development in Africa and marks a significant evolution in educational methodologies, especially in the face of health emergencies like the COVID-19 pandemic, to enhance the preparedness, response, and resilience of healthcare professionals across the continent (2).

Prior to the pandemic, the capacity of the WHO AFRO Emergency Preparedness and Response (3) Learning and Capacity Building Unit for health worker training was limited by logistical challenges and resource constraints, particularly in low-resource settings (4). Traditional in-person workshops and training sessions, though effective, could only reach a limited number of healthcare professionals due to costs, geographical limitations, and the time required to organize and conduct such events. The WHO AFRO Emergency Preparedness and Response (EPR) team typically managed about 30 face-to-face regional trainings annually, engaging 1,500–2,000 participants in total each year.

With the advent of the COVID-19 pandemic, there was a critical need to quickly scale up education and training opportunities and adapt to the constraints imposed by the crisis (5–8). The collaboration between WHO AFRO and Project ECHO transformed this challenge into an opportunity to redesign the learning model. This shift not only aimed to address the immediate needs of the pandemic, but also to lay the groundwork for a more resilient learning system capable of addressing future health emergencies (9). The transition to virtual learning platforms facilitated a dramatic increase in the scope and scale of training efforts (10–15).

This paper argues that the collaboration between WHO AFRO and Project ECHO, initially necessitated by the mobility restrictions and other challenges of the pandemic, has led to a paradigm shift in learning strategies. This shift has broadened the portfolio of learning approaches, placing a greater emphasis on digital learning—both synchronous and asynchronous—while still maintaining a modest component of in-person learning where appropriate. Beyond merely responding to immediate health emergencies, this hybrid learning model equips healthcare workers for a future that demands greater resilience, adaptability, and equity.

## 2 Materials and methods

### 2.1 Clinic design, content, and promotion

From March 2020 until February 2024, WHO AFRO and Project ECHO hosted over 287 digital learning sessions, including six emergency-response series and two non-emergency series (Table 1).

**Table 1:** Summary of WHO Afro series names and characteristics.

**WHO AFRO series name**	**Emergency response or not**	**# of sessions**	**Feedback survey response rate**	**Session time frame**
WHO AFRO IDSR	Emergency	78	6.6%	October 2020–January 2023
WHO AFRO COVID-19 WEBINARS	Emergency	42	30.1%	May 2020–March 2022
WHO AFRO Miscellaneous Emergency	Emergency	41	Survey data not available	February 2021–June 2023
WHO AFRO Pediatric and Adolescent HIV	Non-emergency	29	18.4%	October 2020–October 2023
WHO AFRO Miscellaneous Non-emergency	Non-emergency	23	Survey data not available	June 2020–July 2023
WHO AFRO LAB VCoP	Emergency	14	14.7%	February–October 2021
WHO AFRO ICAN/ACDC IPC	Emergency	12	Survey data not available	July 2022–August 2022
WHO AFRO AVoHC-SURGE	Emergency	7	17.4%	December 2022–May 2023

The structure of WHO/ECHO learning sessions varied based on content. Some were open information sessions, while others included case presentations and experience sharing, primarily targeting clinicians. Additionally, virtual community of practice (VCoP) sessions catered to smaller groups of specialized health professionals needing specific skills or guidelines due to the public health climate in their country or region. Each learning session was live-interpreted and recorded in English, French, and Portuguese to maximize reach and accessibility. ECHO staff and WHO program facilitators met weekly to plan and ensure session quality by optimizing slide decks, camera views, and sound quality. Session recordings were publicly available for asynchronous learning to accommodate busy health professionals.

### 2.2. Data collection and analysis

#### 2.2.1 Cross sectional analysis

This study used cross-sectional and retroactive pre-post designs. Cross-sectional data were collected to assess participation using demographic variables collected via Zoom™ registration. A retroactive pre-post design was employed to evaluate participants' self-reported changes in knowledge or skills, with participants reflecting on their levels before and after the intervention. The latter aimed to provide a more accurate assessment of perceived changes, especially since participants may not have been aware of how much they didn't know before participating in the digital learning sessions. We used a voluntary response sample since the surveys were open to all participants.

Each series used Zoom™ registration questions to gather demographic information about participants, tailored to the specific needs of the program teams. Consistent registration variables included email, country or region, and profession. Zoom™ registration and attendance reports were merged as needed, and session duplicates were removed based on email addresses.

We collected feedback after each session using an anonymous online survey via REDCap (Research Electronic Data Capture) electronic data capture tools hosted at the UNM. REDCap is a secure, web-based software platform designed to support data capture for research studies (16). REDCap surveys included 13 items on one screen, primarily with multiple choice questions (one response option enforced and non-applicable present as appropriate). Once respondents clicked the submit button, they could no longer edit their answers. Survey links were emailed to participants after each session, and completion was linked to a digital certificate of participation as an incentive to increase response rates. Surveys generally closed between 3–4 weeks after each session. This online survey is offered to all international ECHO programs that would like to deploy it. It is an overall assessment that uses CDC-recommended tools. It was designed following the CDC's program evaluation framework's structured and systematic steps, including engaging stakeholders, focusing on the evaluation, ensuring data quality, and guaranteeing that the survey is aligned with general program goals and stakeholder needs (17).

Feedback survey questions included session satisfaction indicators (e.g., whether participants would recommend the session to a colleague), relevance to their work, and knowledge and application outcomes, such as retrospective pre-post knowledge about the session topic, and whether participants would use what they learned in their work. Having been successfully employed in international programs across diverse geographies for over 3 years, the survey has demonstrated its capacity to provide valuable, actionable feedback for program improvement. This long-standing use across various contexts attests to its value and adaptability in capturing meaningful insights.

The evaluation of the programs using Zoom™ registration and REDCap surveys has been approved by the UNM IRB under protocol number 20-469. Per the IRB requirements, surveys included a consent document and a consent question in the introduction. Datasets with personal identifiers were stored in encrypted folders, and only study members listed in the protocol had access. We have reported survey information according to the CHERRIES statement (21).

#### 2.2.2. Cost analysis

Training cost estimate data was collected and analyzed from budget lines, training records, and the interpretation dashboard to assess cost differences between physical and virtual training modalities. The estimated average cost per session and daily cost per participant for virtual training were calculated using expenditure records, interpretation records, and Zoom™ attendance reports. Trends over time were analyzed to identify patterns or changes in attendance and costs associated with both physical and virtual training.

We used descriptive statistics to calculate the number of attendances (unique to each session) the number of attendees (unique to each series) and their respective country counts. For post-session feedback surveys, we focused on the three emergency-response series with the highest response rates, calculated based on the number of submitted survey entries divided by total attendance (Table 1). Descriptive statistics were used to analyze session relevance and application of learnings, and the Wilcoxon rank sum test was used for paired data analysis of pre-and post-session knowledge levels. We reviewed all submissions, even if the responses to specific questions were missing. Given that feedback surveys were anonymous, we could not fully deduplicate the datasets. However, 93% of the WHO COVID-19 Webinar survey respondents, 97% of WHO AVoCH -SURGE Webinars, and 96% of WHO LAB Virtual Community of Practice (VCoP) sessions, shared their emails at the end of the survey to receive certificates. Based on those, we identified and excluded duplicate entries before conducting statistical analysis. Respectively, for the three programs outlined above, we excluded 7% of the records for the first two (393 out of 5,382 and 34 out of 466) and 6% (8/140) for the last. This analysis was conducted using R (18), RStudio (19), and the MASS package (20).

### 2.3. Data limitations

Since 2020, there has been significant variation in questions and response choices between learning sessions, limiting the ability to compare across sessions and series. However, we were able to analyze three WHO/ECHO Series attendance/registration reports and feedback surveys with the highest response rates over the last 4 years. These included WHO COVID-19 Webinars, WHO AVoCH -SURGE Webinars, and WHO LAB Virtual Community of Practice (VCoP) Sessions. As outlined previously, given that feedback surveys were anonymous, we could not deduplicate the dataset fully. VCoP sessions, targeting specific learners, often had fewer attendances than webinars, but featured higher engagement through smaller interactive group discussion. Finally, these results are based on a sample with potential bias due to low response rates.

## 3 Results

### 3.1 Participant survey

**Figure 1:**
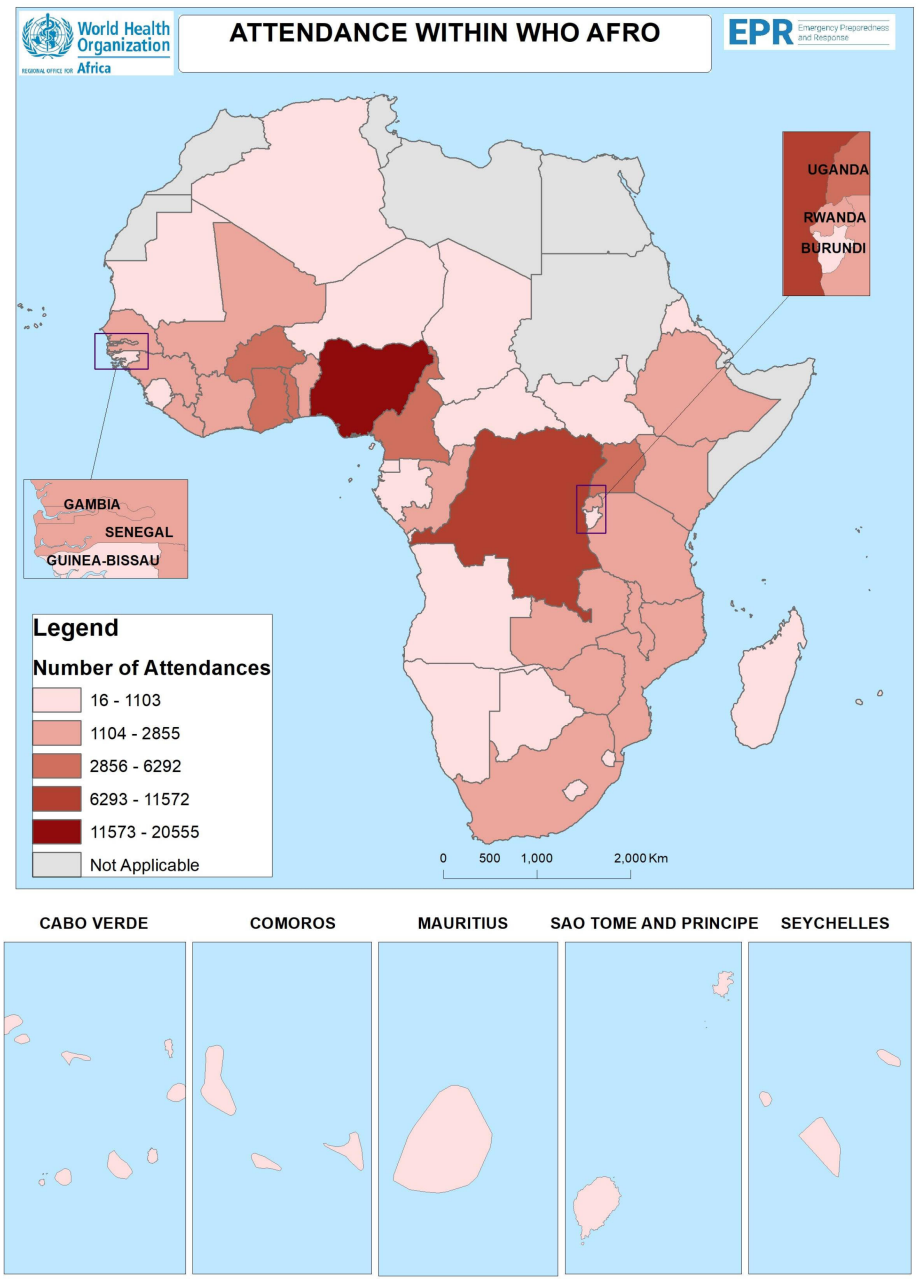
Concentration of attendees by countries in the African region. Situation as of: 30 April 2024. Data Source: MOH in WHO AFRO. Emergency Preparedness and Response. Regional office for Africa. World Health Organization© WHO 2024. All rights reserved. The boundaries and names shown and the designations used on this map do not imply the expression of any opinion whatsoever on the part of the World Health Organization concerning the legal status of any country, territory, city or area or of its authorities, or concerning the delimitation of its frontiers or boundaries.

WHO AFRO and Project ECHO hosted six emergency-response series and two public health topical learning series from March 2020 to February 2024, with a total of 125,816 participants from 173 countries. Participants from the 54 African countries totaled 100,053, with 79.5% (79,542 participants) from the 47 WHO AFRO countries. Nigeria had the highest number of participants (20,555), followed by the Democratic Republic of Congo (11,572) and Ghana (6,292; Figure 1).

Pre-post retrospective knowledge of the session topic was measured in post-session surveys with before and after questions for respondents to subjectively rate their knowledge. The paired data for the three series outlined above showed that before the webinars 29.4% of participants considered themselves “Not at all/slightly knowledgeable,” 24.9% as “Very knowledgeable,” and 4.5% as “Extremely knowledgeable.” However, post-webinar assessments revealed a significant increase in knowledge levels: only 1.7% remained in the “Not at all/slightly knowledgeable” category, while 64.2% were now “Very knowledgeable,” and 18.7% were “Extremely knowledgeable.” The *p*-value is < 0.001, indicating a statistically significant improvement. These results were consistent across all topics (Table 2).

**Table 2:** Distribution of attendees by knowledge and topics.

**Topic**	**Number (*N*)**	**Category**	**Pre training**	**Post training**
General	5,350	Not at all/slightly knowledgeable	29.4	1.7^*^
		Moderately knowledgeable	41.2	15.4
		Very knowledgeable	24.9	64.2
		Extremely knowledgeable	4.5	18.7
COVID-19	4,789	Not at all/slightly knowledgeable	26.9	1.8^*^
		Moderately Knowledgeable	43.3	13.2
		Very knowledgeable	25.1	66.6
		Extremely knowledgeable	4.7	18.4
AVoHC	430	Not at all/slightly knowledgeable	46.7	0.7^*^
		Moderately knowledgeable	27.4	32.3
		Very knowledgeable	23	47.9
		Extremely knowledgeable	2.8	19.1
Lab	131	Not at all/slightly knowledgeable	63.4	0.8^*^
		Moderately knowledgeable	9.9	42.7
		Very knowledgeable	22.1	31.3
		Extremely knowledgeable	4.6	25.2

^*^p value < 0.001.

**Figure 2:**
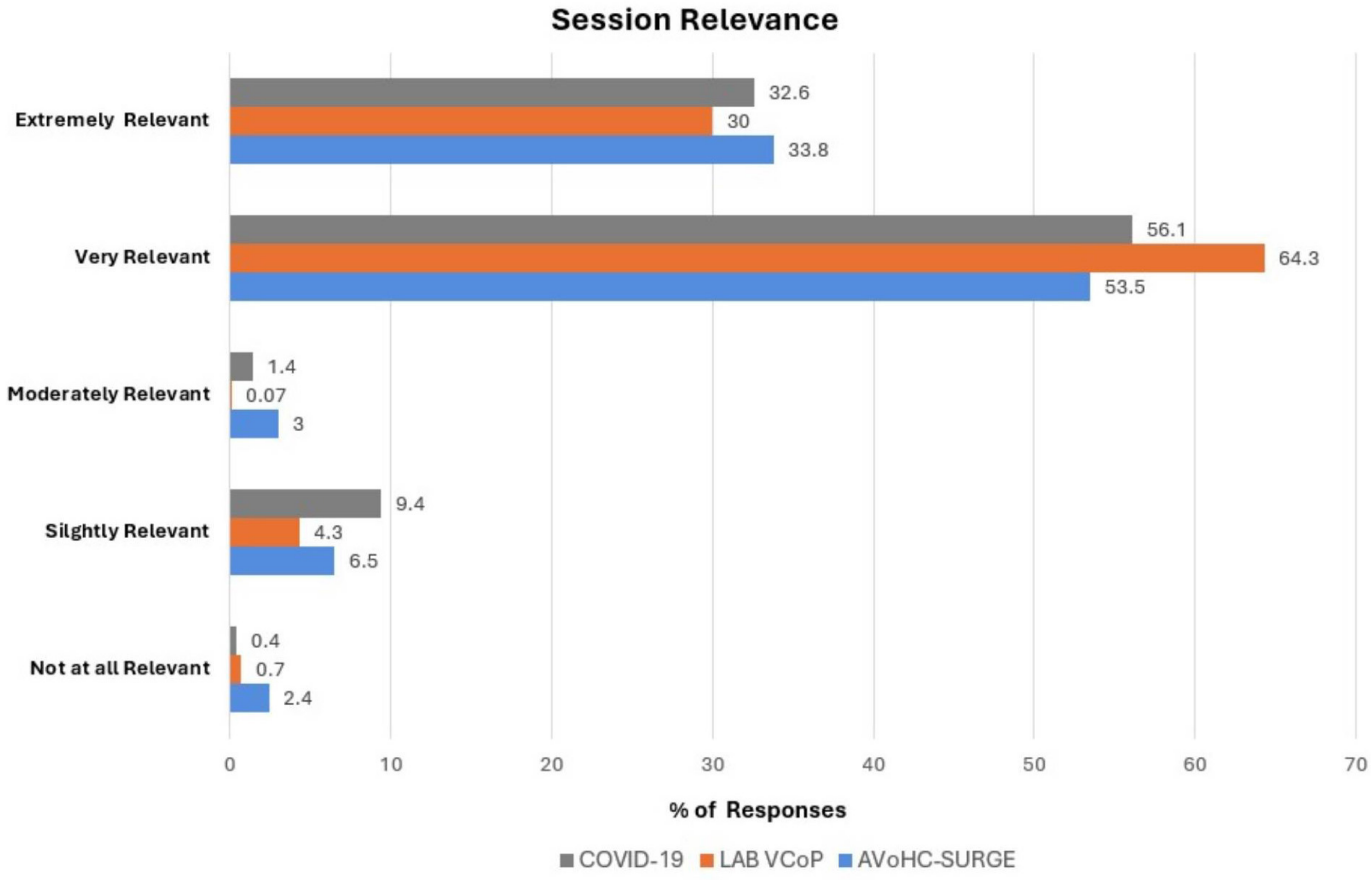
Distribution of attendees by perceived relevance of training session on their work.

Participants expressed varying perceptions of the learning session's relevance to their work, however, more than 85% found the learning sessions to be very or extremely relevant independent of the series. For those, the Lab Virtual Community of Practice (VCoP) training had the highest ratings for relevance to participant's work (94.3%). AVoCH_SURGE and COVID-19 learning series had similar relevance ratings, respectively 87.3 and 88.7% (Figure 2).

**Figure 3:**
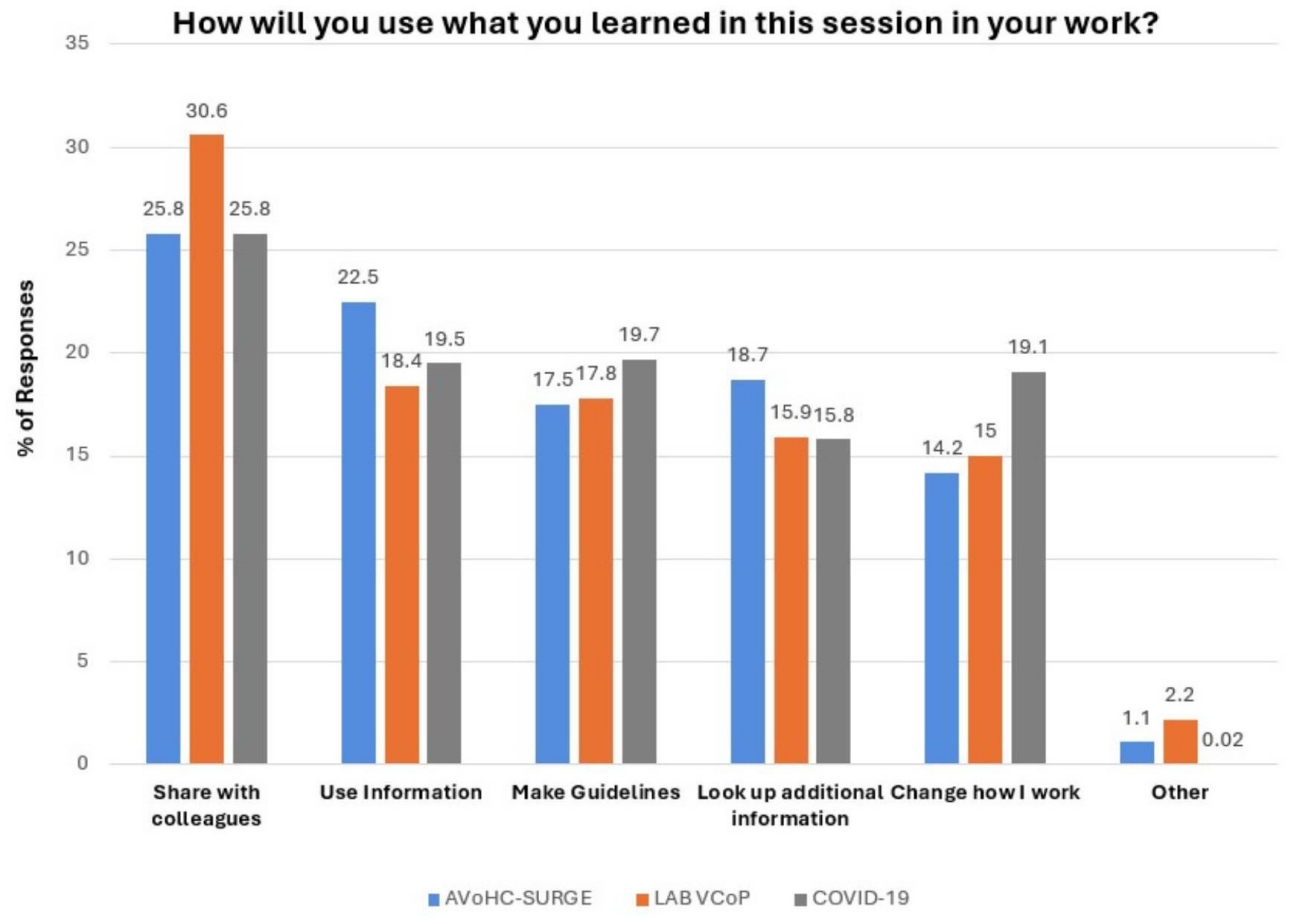
Distribution of attendees by ways in which they will use what is learned.

For WHO AFRO LAB VCoP sessions, WHO AFRO AVoHC-SURGE Webinars, and COVID-19 webinars, participants were asked how they planned to use what they learned. Sharing learnings with colleagues was the most common option selected by 30.6% from the WHO AFRO LAB VCoP, 25.8% from WHO AFRO AVoHC-SURGE, and 25.8% from COVID-19 webinars (Figure 3).

### 3.2 Training cost estimation

#### 3.2.1 In-person training cost estimate (2018–2019)

Between 2018 and 2019, WHO AFRO EPR conducted five in-person training sessions focused on Integrated Disease Surveillance and Response (IDSR), each lasting an average of 5.5 days, plus two travel days. These training sessions averaged 28.6 participants each. The financial commitment was substantial, with total expenditures amounting to $444,471. Individual training event costs ranged from $37,860 to $135,000. The direct cost to the organization ranged from $440 to $757 per participant per day, averaging $612 daily ($76.5 per hour) per participant, or $3,266 per participant for the 5.5-day training.

#### 3.2.2 Virtual training cost estimate (2021–2023)

To adapt to new norms and reduce costs, WHO AFRO EPR transitioned to virtual training from 2021 through 2023. This change dramatically reduced the cost per participant to between $3 and $4 per hour and significantly increased accessibility. Consequently, average attendance surged to 404 participants per learning session. This marked increase highlights the scalability and cost-efficiency of virtual training, proving it to be a formidable alternative to traditional in-person learning sessions. This model was particularly beneficial to participants in regions where access to such training was previously limited or non-existent.

## 4 Discussion

### 4.1 Democratizing access to WHO technical expertise

The virtual learning approach expanded access to learning and enhanced the preparedness, response, and resilience of healthcare professionals across the continent, marking a significant evolution in educational methodologies during global health emergencies. Before COVID-19, face-to-face training aimed to disseminate WHO AFRO EPR's technical expertise, but reached only a select group (~2,000 per year) who could overcome logistical, geographic, and financial challenges. The shift to virtual learning democratized access by greatly reducing these barriers (22, 23); only a Wi-Fi or data-connected device was required. From 2020–2023, there were 125,816 attendances from 173 countries and regions, over 15 times the pre-COVID-19 pandemic in-person rate. This increase was primarily due to African participation, with 79.52% (100,053) of participants joining from the 47 WHO AFRO member states.

### 4.2 Cost- and time-efficient approach

The increased participation in WHO AFRO webinars, as outlined in the results section, is directly related to the cost and time efficiency of these learning opportunities being virtual. Virtual platforms eliminate geographical barriers, enabling healthcare professionals to access crucial health information globally without costly and time-consuming travel, and without requiring participants to leave their work posts. Traditional in-person training programs entail significant expenditures, including travel, accommodation, venue rentals, and printed materials. The significant cost savings from reduced travel and accommodation expenses allow the WHO AFRO to allocate resources more efficiently, thereby expanding the reach and frequency of their virtual learning sessions.

Additionally, virtual learning facilitates the rapid and widespread dissemination of up-to-date health information and training, crucial in emergencies like the COVID-19 pandemic. The scalability of virtual platforms enables WHO to reach a larger audience without the logistical challenges of in-person training sessions. This efficiency not only lowers per-participant costs, but also accelerates the process of knowledge and best practice transfer, ensuring healthcare workers worldwide are well-equipped with the latest guidelines and practices. Consequently, virtual learning enhances WHO's capacity to improve global health outcomes in a cost-effective and timely manner. This collective enhancement of knowledge and best practices directly contributes to improved health outcomes globally (14, 24), demonstrating how the cost and time efficiency of virtual learning fosters increased participation and amplifies its impact.

### 4.3 Bidirectional learning

Bidirectional learning, the cornerstone of WHO AFRO EPR's learning strategy, has transformed knowledge sharing and action-taking through dynamic exchanges via Q&A sessions and participant feedback. By using feedback to identify learning needs, we have tailored our learning programs to address specific gaps and challenges, ensuring the content remains relevant and impactful. Real-time feedback has allowed us to swiftly revise session content, keeping our learning content practical and current. Digital learning sessions have broadened participation, bringing in diverse perspectives that enrich the learning experience and foster a holistic approach to health emergency preparedness and response. For instance, the IDSR VCoP was developed in response to participant expressed needs and feedback, exemplifying the impact of bidirectional learning.

### 4.4 Call for an evidence-based approach to digital learning

In collaboration with WHO AFRO EPR, Project ECHO teams at the UNM ECHO Institute and the ECHO India Trust applied insights from more than 15 years of experience implementing the evidence-based Project ECHO case-based tele-mentoring model to the webinar format. Virtual learning sessions also routinely employed other adult learning best practices, such as polling and ample Q&A to maximize interactivity (25, 26). Now that the initial pandemic response has waned, there is an opportunity to assess gaps in the evidence (27).

First, there is a need to standardize participant and feedback data collection to enable more cross-program analysis. The reduced financial and personal-life burdens (e.g. time away from family and the need to secure childcare while traveling) of virtual options suggest that the virtual learning sessions are more accessible and convenient to women and early-career professionals from a wider variety of healthcare professions (28). However, analysis of categories such as participant gender, career level, and professional category is currently limited due to incomplete or inconsistent data collection. More in-depth post-session and longitudinal evaluation is needed to better understand the impact programs have on practice change.

Second, further research is needed to determine the optimal dose, including frequency and duration of digital learning sessions, to maximize learning outcomes, equity, and resource utilization. Questions include whether biweekly sessions are as effective as weekly or monthly ones, how one-time learning sessions or limited series webinars compare to ongoing virtual communities of practice, and which learning competencies and outcomes are best suited for virtual vs. in-person learning. Identifying barriers to accessing learning opportunities and implementing learnings is also essential. While professional experience suggests a balance between virtual asynchronous, virtual synchronous, and in-person synchronous learning is important, there is limited peer-reviewed data on tailoring these approaches to specific learning objectives.

Third, research into the cost-effectiveness of digital learning initiatives, considering time, financial, and carbon emissions costs, is necessary (29–31). While there is some data on cost-effectiveness from the implementer perspective, there is limited information on the cost-effectiveness for participants and health systems when virtual learning outcomes are linked to patient outcomes and cost-savings. The negative impact of carbon emissions on the climate crisis increases the importance of measuring and factoring in carbon emission costs to the cost of education and training opportunities. In-person learning opportunities are associated with higher carbon emissions due to participant air and ground travel (which accounts for 4–5% of global emissions annually), short-term accommodations, and single-use items like conference booklets, lanyards, posters, and beverage containers (32). While some data supports virtual learning as a lower-emission option, the specific emission impact of these programs remains unknown.

### 4.5 Implications of the study: a new normal in learning

The COVID-19 pandemic necessitated a rapid switch to an exclusively digital learning program, democratizing access to WHO technical expertise and allowing broader participation from diverse geographies. Participant feedback data shows that these early digital learning initiatives extended beyond achieving scale and increasing equity of access to just-in-time learning to also achieving significant impact.

While the evidence base for virtual learning is still being built, this paper advocates not for a “digital-only” future, but a “digital-first” new normal. In this digital-first approach, trainers prioritize digital learning when appropriate while utilizing a variety of tools (asynchronous online courses, webinars, virtual face-to-face, in-person). This hybrid approach leverages a diverse learning ecosystem offering a flexible and dynamic experience. Travel for in-person training would be reserved for specific, targeted objectives and may be preceded by prerequisite virtual engagements and/or followed by ongoing virtual learning opportunities.

The pandemic highlighted the importance of leveraging emerging technology to build and maintain effective learning programs. The new normal retains the spirit of innovation from early COVID-19 virtual learning, embracing the latest technologies and methodologies, including artificial intelligence and adult learning theory, and can also be useful for future pandemics (33, 34).

The new normal seeks to integrate digital learning into standard practices without compromising the efficiency, greater reach and increased equity of access achieved during the pandemic. Training sessions can be planned and implemented rapidly to disseminate information quickly to a diverse audience. This approach embraces quality improvement by integrating lessons learned from after-action reviews, providing consistent training on digital platforms, and securing increased financial and human resources for virtual learning.

## 5 Conclusion

The partnership between WHO AFRO EPR and Project ECHO not only helped navigate the challenges posed by COVID-19, but also laid the groundwork for a robust “new normal” in health workforce education, training, and continuing professional development. The new normal demonstrates that virtual training can facilitate substantial knowledge acquisition among a broad audience at a global level. This innovative learning approach, combined with the ECHO “all teach, all learn” case-based learning model and informed by continuous assessments, ensures ongoing preparedness and offers valuable lessons for future healthcare education endeavors. As the COVID-19 response evolves, the initiative's focus on democratizing knowledge through interactive and accessible learning sessions continues to empower public health leaders and frontline health workers, offering them more equitable access to lifesaving knowledge and best practices.

The collaboration between WHO AFRO EPR and Project ECHO represents a significant advancement in healthcare education, setting a precedent for future continuing professional development programs. By leveraging digital technology and fostering an “all-teach, all-learn” model, this initiative has not only responded adeptly to the immediate needs catalyzed by the COVID-19 pandemic, but has also laid the groundwork for a more resilient, inclusive, and effective approach to healthcare worker education and training worldwide. To sustain this momentum and further expand access to critical health knowledge, stakeholders—including governments, donors, and health institutions—must commit to continued investment in digital learning infrastructure, training, and technology.

## Data Availability

The raw data supporting the conclusions of this article will be made available by the authors, without undue reservation.
